# MR-guided adaptive stereotactic body radiotherapy (SBRT) of primary tumor for pain control in metastatic pancreatic ductal adenocarcinoma (mPDAC): an open randomized, multicentric, parallel group clinical trial (MASPAC)

**DOI:** 10.1186/s13014-022-01988-6

**Published:** 2022-01-25

**Authors:** M. Pavic, M. Niyazi, L. Wilke, S. Corradini, M. Vornhülz, U. Mansmann, A. Al Tawil, R. Fritsch, J. Hörner-Rieber, J. Debus, M. Guckenberger, C. Belka, J. Mayerle, G. Beyer

**Affiliations:** 1 Department of Radiation Oncology, University Hospital Zurich and University Zurich, Rämistrasse 100, 8091 Zurich, Switzerland; 2 Department of Radiation Oncology, University Hospital, LMU, Marchioninistraße 15, 81377 Munich, Germany; 3 Department of Medicine II, University Hospital, LMU, Munich, Germany; 4 Institute for Medical Information Processing, Biometry, and Epidemiology, LMU, Munich, Germany; 5 Department of Medical Oncology, University Hospital Zurich and University Zurich, Rämistrasse 100, 8091 Zurich, Switzerland; 6 Department of Radiation Oncology, Heidelberg University Hospital, Im Neuenheimer Feld 400, 69120 Heidelberg, Germany; 7 National Center for Radiation Oncology (NCRO), Heidelberg Institute for Radiation Oncology (HIRO), Im Neuenheimer Feld 400, 69120 Heidelberg, Germany; 8 Bavarian Cancer Research Center (BZKF), Munich, Germany

**Keywords:** Stereotactic body radiotherapy, SBRT, Pancreatic cancer, Metastasized, MR-guided radiotherapy, Pain control, Quality of life

## Abstract

**Background:**

Pain symptoms in the upper abdomen and back are prevalent in 80% of patients with metastatic pancreatic ductal adenocarcinoma (mPDAC), where the current standard treatment is a systemic therapy consisting of at least doublet-chemotherapy for fit patients. Palliative low-dose radiotherapy is a well-established local treatment option but there is some evidence for a better and longer pain response after a dose-intensified radiotherapy of the primary pancreatic cancer (pPCa). Stereotactic body radiation therapy (SBRT) can deliver high radiation doses in few fractions, therefore reducing chemotherapy-free intervals. However, prospective data on pain control after SBRT of pPCa is very limited. Therefore, we aim to investigate the impact of SBRT on pain control in patients with mPDAC in a prospective trial.

**Methods:**

This is a prospective, double-arm, randomized controlled, international multicenter study testing the added benefit of MR-guided adaptive SBRT of the pPca embedded between standard of care-chemotherapy (SoC-CT) cycles for pain control and prevention of pain in patients with mPDAC. 92 patients with histologically proven mPDAC and at least stable disease after initial 8 weeks of SoC-CT will be eligible for the trial and 1:1 randomized in 3 centers in Germany and Switzerland to either experimental arm A, receiving MR-guided SBRT of the pPCa with 5 × 6.6 Gy at 80% isodose with continuation of SoC-CT thereafter, or control arm B, continuing SoC-CT without SBRT. Daily MR-guided plan adaptation intents to achieve good target coverage, while simultaneously minimizing dose to organs at risk. Patients will be followed up for minimum 6 and maximum of 18 months. The primary endpoint of the study is the “mean cumulative pain index” rated every 4 weeks until death or end of study using numeric rating scale.

**Discussion:**

An adequate long-term control of pain symptoms in patients with mPDAC is an unmet clinical need. Despite improvements in systemic treatment, local complications due to pPCa remain a clinical challenge. We hypothesize that patients with mPDAC will benefit from a local treatment of the pPCa by MR-guided SBRT in terms of a durable pain control with a simultaneously favorable safe toxicity profile translating into an improvement of quality-of-life.

***Trial registration*:**

German Registry for Clinical Trials (DRKS): DRKS00025801. Meanwhile the study is also registered at ClinicalTrials.gov with the Identifier: NCT05114213.

## Background

Pancreatic cancer is the third leading cause of cancer-related death in the United States and Europe, but comes at 9th place in incidence, reflecting the high burden and lethality of this cancer type [1, 2]. The incidence of pancreatic cancer is rising worldwide [3], increasing the number of patients living with pancreatic carcinoma. A significant proportion of patients (about 40%) presents with metastatic disease at diagnosis. These patients often present with symptoms such as abdominal pain due to perineural invasion of the primary tumor, weight loss and symptoms of duodenal and/or bile obstruction [4]. The aforementioned symptoms are reported as primary symptoms in about 50–60% of patients with newly diagnosed pancreatic carcinoma [5]. In advanced cancer, up to 80% of patients are known to suffer from pain [6]. Currently, the gold standard for treatment of metastatic pancreatic ductal adenocarcinoma (mPDAC) is systemic therapy [7, 8]. Although recent advances in chemotherapeutic regimens achieved a moderate improvement of life expectancy [9–11], the overall survival of mPDAC patients remains low, asking for a more focus on quality of life and symptom control.

Adequate pain control is often difficult to achieve due to the infiltrative growth of the primary tumor and the limited treatment options. Chemotherapy itself was shown to have an impact on pain with response rates up to 67% [12]. However, only one single trial reported such a high response rate without detailed information on pain assessment. Most remaining trials report pain relief in about 20–30% of patients. Usually, the use of opioids is required, and continuous intake is frequently associated with relevant side effects. Alternative treatment options for patients, insufficiently responding to pain medications, are celiac plexus block, radiofrequency ablation (RFA), irreversible electroporation (IRE), high intensity focused ultrasound (HIFU) and radiation treatment [13]. In a Cochrane meta-analysis, celiac plexus block shows a significant, but small effect on pain control [14]. Pain reduction is rather short-lasting, as reported by a systematic review [15]. All these local procedures may offer temporary symptom relief. In contrast to the other procedures, radiotherapy has the advantage of being non-invasive and having a favourable effect on quality of life [13].

Conventional short-course radiotherapy, a well-established treatment option for cancer-related pain [16], has been investigated in few studies for pain management in pancreatic cancer. Most studies were of retrospective nature and conducted in a population with a short median overall survival of only 3–5 months. Those studies reported a shortly lasting pain response in the majority of patients [17, 18]. In a retrospective evaluation, patients experienced pain relief when treated with 1–3 fractions of 8 Gy each, of whom 7% reported complete pain relief (numeric rating scale (NRS) of 0). Pain relief started 1 week after the end of treatment. Its median duration was 2.5 months in a cohort with poor median survival of 3.5 months. Interestingly, patients that showed a treatment response did experience a significant prolonged median OS of 4.4 months versus 1.7 months [18]. With improvement of systemic therapy and increase of overall survival time, a persistent symptom control becomes more important. Long-term conventional fractionated radiotherapy with delivery of 40–60 Gy to the primary tumor leads to higher pain response rates with pain relief in up to 100% of patients and high complete response rates [19, 20]. A major drawback of this treatment regimen is its long duration and therefore a long chemotherapy-free interval, which is the most crucial treatment component in this patient cohort with regard to survival.

The aforementioned paves the way for stereotactic body radiotherapy (SBRT), where escalated radiation doses are delivered in few ambulatory treatment fractions and can therefore be seamlessly integrated into full-dose systemic therapy regimens. SBRT has proven to be an efficacious and cost-effective [21] treatment in patients with locally advanced pancreatic cancer (LAPC) providing 1-year local-control rates of 72.3% in a systematic review [22]. There is some evidence that SBRT leads to a better local control compared to conventionally fractionated radiation therapy [23]. When applied in 5 fractions, it has a favorable toxicity profile [24]. In a prospective trial, SBRT did also show a significant decrease of the pancreatic pain score (measured by the QLQ-PAN26) 4 weeks after treatment [24]. The capacity of SBRT to relieve pain symptoms was confirmed by two small retrospective series on SBRT for elderly or medically inoperable patients reporting pain response rates of 73–80% [25, 26] and a systemic review with an overall pain relief rate of 84.9% [27].

So far, prospective data on pain control after SBRT is limited. Additionally, evidence about SBRT for primary pancreatic tumor in metastatic patients is very scarce and mainly of retrospective nature. This indicates the need for a well-designed prospective trial with thorough evaluation of pain symptoms and its outcome after SBRT of the primary tumor. Therefore, we aim to investigate the impact of SBRT to the primary tumor in metastasized pancreatic cancer patients on pain control in a prospective randomized trial. SBRT will be performed using a MR-LINAC platform, a hybrid machine combining a linear accelerator (LINAC) with a MRI scanner allowing for on-table MR imaging with gated treatment and daily plan adaptions based on the current anatomy. This technology enables to deliver ablative doses, while maintaining the risk of toxicity to the least possible level.

## Methods/design

### Aim and study design

**Fig. 1:**
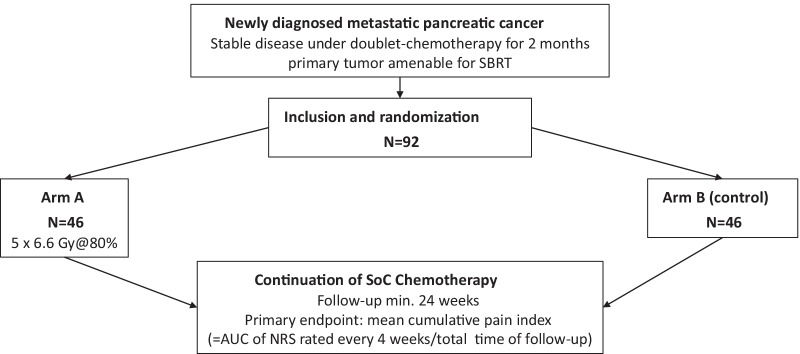
Study flowchart

MASPAC trial (DRKS 00025801, NCT05114213) is a prospective, double-arm, parallel group, randomized controlled, international multi-center study. The trial aims to investigate the benefit of MR-guided SBRT of the primary pancreatic tumor on pain control and prevention of pain in patients with histologically proven mPDAC after an initial course of 8 weeks of standard-of-care palliative chemotherapy (SoC-CT). Only patients with at least stable disease in the restaging after 8 weeks of SoC-CT will be included into the study. Those with progressive disease will be considered screening failures and therefore not eligible for the study. Patients included into the trial are 1:1 randomized to either Arm A, receiving MR-guided SBRT of the primary tumor with continuation of SoC-CT thereafter without any relevant delay (experimental arm), or Arm B, continuing SoC-CT without SBRT of the primary tumor (control arm). Chemotherapy is given in both arms, A and B, according to the current standard of care and at the decision of the treating medical oncologist, consisting of at least a doublet-chemotherapy, and excluding monotherapy regimens. Patients will be followed up for a minimum of 6 months and a maximum of 18 months. Figure 1 summarizes the study flow of patients within the trial.

## Primary and secondary endpoints

As outlined above, we aim to primarily test the efficacy of additional SBRT on pain control. Therefore, the primary endpoint of the study is the “mean cumulative pain index” (MCPI); calculated as the area under the curve (AUC) of pain scores, rated every 4 weeks until death or end of follow-up using the NRS with values from 0 (= no pain) to 10 (= worst pain). Pain will be assessed as patient reported outcome and indirectly derived from pain medication used by the patient. The AUC results from joining the point s (= time of examination, NRS value measured) over the individual follow-up time, divided by the total follow-up time. It is anticipated that intercurrent events (such as loss to follow-up, death, etc.) need to be considered for a reliable interpretation of the primary endpoint.

Secondary endpoints include average pain on NRS at 24 weeks, time interval until definitive deterioration (= increase of average pain on NRS of 3 points over baseline sustained over 4 weeks), biliary complications, defined as cholangitis or post-hepatic cholestasis requiring drainage or stenting, nutritional status measuring bioimpedance-derived phase angle (BIA) every 12 weeks and death from any cause. Further secondary endpoints are treatment toxicity according to CTCAE v5.0 and quality of life (QoL) assessment using the Euro-QoL 5 dimensions questionnaire (EQ-5D-5L) and FACT-Hepatobiliary Symptom Index—(FHSI-8).

### Patient selection

A sample size of 92 patients is aimed to be recruited. The enrollment period is planned for 24 months with a minimum follow-up of 6 months and a maximum follow-up of 18 months. Participating centers are three university hospitals in Germany and Switzerland (Munich, Zurich and Heidelberg). The inclusion and exclusion criteria are outlined in Table 1.

**Table 1:** In- and exclusion criteria

Inclusion criteria	Exclusion criteria
Male and female patients with histologically proven, metastatic pancreatic adenocarcinoma of the pancreatic head or body amenable for MR-guided adaptive SBRT with at least stable disease after 8 weeks of standard of care doublet chemotherapy age > 18 years Eastern Cooperative Oncology Group (ECOG) Performance Status 0, 1 or 2 Ability to follow study instructions and likely to attend and complete all required visits Written informed consent of the subject	Progressive disease after 8 weeks of SoC-CT Subjects not able to give consent Subjects without legal capacity who are unable to understand the nature, scope, significance and consequences of this clinical study Simultaneous participation in another clinical study or participation in any clinical trial involving an investigational medicinal product or treatment within 30 days prior to beginning of this study Subjects with a physical or psychiatric condition which at the investigator’s discretion may put the subject at risk, may confound the study results, or may interfere with the subject’s participation in this study Women of child bearing potential or sexually active males not willing to use effective contraception while on treatment and 12 weeks after the end of treatment (such as oral, injectable, or implantable contraceptives, or intrauterine contraceptive devices) unless they are surgically sterilized/hysterectomized or there are any other criteria considered sufficiently reliable by the investigator in individual cases Previous pancreatic surgery Previous abdominal radiotherapy of the upper abdomen ECOG > 2 Patients with contraindications for MR imaging (e.g. non-MRI-compatible cardiac pacemaker or ICD/Cochlea implant/metal implants/severe claustrophobia) Patients with contraindications for doublet-chemotherapy Patients with contraindications for MR-guided adaptive SBRT Biopsy proven tumor invasion into the stomach and/or duodenum Concomitant non-pancreatic malignancy. Patients being treated for a non-pancreatic malignancy with tumor control for over 3 years are eligible Medically uncontrolled pain

### Study intervention/treatment and procedures

Patients found eligible for study participation and from whom written informed consent was obtained during screening period, will be included. Patients will be registered through a computer system and a study-ID will be assigned by the institute for biometrics and epidemiology (IBE) in Munich via the same computer system with stratification according to age and ECOG.

In arm A, the primary tumor is treated with SBRT in 5 fractions on a MR-LINAC platform in breath-hold technique. The MR-LINAC is a CE-mark approved medical device and already in use in clinical routine. It integrates MR imaging and a LINAC into a hybrid system allowing for on-table radiotherapy plan adaptation according to the daily anatomy. Daily adaptive planning is performed aiming to maintain stringent dose constraints for organs at risk (OAR), such as the duodenum, stomach, bowel, liver, kidneys and the spinal cord, while trying to give the full dose of planned 6.6 Gy prescribed at the 80% isodose level to the target volume. SBRT will be performed in a few days straight after randomization into arm A between two cycles of SoC-CT, but not concurrently to chemotherapy.

All patients in study arm A and B will rate their pain in the abdominal region on a NRS and report their concomitant medication at baseline and all 4 weeks thereafter. They will undergo at baseline, 4 and 12 weeks after baseline and every 12 weeks thereafter a more intensive assessment with physical examination, laboratory values, body impedance analysis (BIA) in order to monitor nutritional status, examination and reporting of adverse events as well as of QoL via answering questionnaires and answer the Brief pain inventory. Review of imaging will be performed 12 weeks after randomization and then every 12 weeks thereafter. Additionally, blood collection will be performed at randomization and 4 weeks and 24 weeks after that to evaluate predictors of treatment response using deep immune phenotyping, metabolomics and whole blood transcriptomics. Table 2 lists all the study planned study procedures including screening activities.

**Table 2:** Schedule of activites

Procedures	Screening and inclusion	Baseline^6^	MR-guided adaptive SBRT (Arm A only)	Week 4 post randomization	Every 4 weeks post randomization^1^	Every 3 months post randomization	Study termination (18 months post randomization)
Informed consent, demographic data, in-/exclusion criteria, medical history	√						
Physical examination^9^		√	√^2^	√		√	√
Laboratory assessment^8^		√		√		√	√
Study specific blood and urine collection^7^		√		√		√^3^	
Pain assessment^10^		√		√	√^4^	√^5^	
Assessment of biliary complications^11^		√		√		√	√
Assessment of nutritional status^12^		√		√		√	√
Assessment of quality of life^13^		√		√		√	√
Randomization		√				√	√
Review of imaging	√					√	√
MR-guided adaptive SBRT			√				
Concomitant disease	√			√		√	√
Concomitant medication	√		√	√	√	√	√
Adverse events and toxicity			√	√		√	√

^1^Either during clinical visit or phone call

^2^Vital signs only

^3^Only week 24

^4^NRS

^5^NRS and Brief Pain Inventory

^6^Study specific examinations and blood withdrawal should only be performed after having obtained written informed consent from the patient and before randomization of the patient

^7^Study specific blood withdrawal for exploratory objectives will be conducted in conjunction with clinically indicated routine blood draws

^8^Routine laboratory: full blood count, differential blood count, aPTT, INR, Sodium, Potassium, Creatinine, serum urea, Bilirubin (total and conjugated), ASAT, ALAT, gGT, AP, Lipase, CRP, LDH, CA19.9, Albumin, Phosphate, Magnesium, Calcium, 25-OH-Vitamin D, HbA1c. The pregnancy test: bHCG urine dip stick (only in women of child bearing age at baseline) should be performed at baseline and the result should be known before randomization

^9^Physical examination includes: clinical investigation of skin, chest and abdomen; vital signs: blood pressure, pulse, temperature, breath rate, oxygen saturation; ECOG by physician or trained specialized nurse

^10^Pain assessment: Brief pain Inventory questionnaire, intensity assessed by NRS

^11^Biliary complications: cholangitis, post-hepatic cholestasis requiring biliary stenting or PTCD

^12^Nutritional status: BMI, BIA

^13^Quality of life: EQ-5D-5L and Fact Hepatobiliary Symptom Index FHSI-8

### Safety, adverse events and quality assurance

Any adverse event (AE) occurring between the visit with the first intervention to the subject and the last visit with the last individual specific examination of the subject will be documented and evaluated in the electronic case report form (eCRF). Every unforeseen or unfavorable medical occurrence in a patient including any abnormal sign (e.g. abnormal physical exam or laboratory finding), symptom, or disease, temporally associated with the patients’ involvement in the clinical study, whether or not considered related to participation in the clinical study is defined as an adverse event. Seriousness, intensity (Grade 0–5) and causality to the study intervention (not related, unlikely, possibly related, probably related, definitely related) will be determined. During the clinical study, quality control and quality assurance will be ensured through central monitoring.

### Study discontinuation

Whenever a subject is withdrawn from the study, the circumstances of the withdrawal or discontinuation will be recorded in detail in the CRF (patient wish/voluntarily, adverse event, other reasons) and a complete final examination as scheduled for the termination visit should be conducted.

If a subject does not return for a scheduled visit, every effort should be made to contact the subject (also regarding any unresolved adverse events).

In any circumstance, every effort should be made to document subject outcome, if possible. In case of a dropout due to an adverse event, the subjects should be monitored outside the trial as long as medically indicated.

### Ethical conduct

The study will be conducted in accordance with the provisions of the Declaration of Helsinki, the International Conference on Harmonization “Good Clinical Practices,” and the respective national regulations.

Participating centers have to provide written approval of the institutional medical ethics committee. The study was approved by the ethics committee at the leading center in Munich (Project Number 20-0973), in Zurich (BASEC-Nr. 2021-01427) and in Heidelberg.

### Patient participation

Two patient support groups, “Arbeitskreis der Pankreatektomierten” (https://www.bauchspeicheldruese-pankreas-selbsthilfe.de/) and “Deutsche Pankreashilfe” (https://www.pankreashilfe.de/), have been introduced to the trial concept and were given the opportunity to contribute to protocol and outcome measures. The content of this trial will be presented to these patient self-help groups and will be made available online as soon as ethics approval has been granted.

### Statistical analysis

The study examines the additional benefit of MR-guided SBRT of the primary tumor, embedded in standard chemotherapy, for pain control and prevention of pain in patients with metastatic PDAC in a 1:1 randomized fashion.

The primary endpoint is the MCPI (normalized Mean Cumulative Pain Index over 4 weeks) which is formally defined as the cumulative pain index calculated via the trapezoid rule over the complete time of follow-up (AUC) and divided by the number of the observed 4 weeks periods. A simple example is given below. At present, no data is available to perform an informed sample size calculation for our study. We also do not have access to documented longitudinal pain data in a comparable population. For an approximative sample size guess we used pre- and post- data on pain over a period of 6 months reported by Gourgou et al. [28].

The calculation of the endpoint over the 24 weeks uses AUC = (W_0_ + W_24_) · 0.5 · 24.

The MCPI over 24 weeks (24 = 6 · 4) is given by MCPI = AUC/6 = (W_0_ + W_24_) · 0.5 · 24/6 = (W_0_ + W_24_) · 2.

The pain measurement data are as follows:

Group I: W_0_ = 43.9 at baseline (σ_0_ = 30.7 Std)
  W_24_ = 19.3 after 24 weeks FU (σ_24_=25.0 Std)
  MCPI: (43.9+19.3) · 2 = 126.4 Std: 111.4
Group II: W_0_ = 46.3 at baseline (σ_0_=30.5 Std)
  W_24_ = 26.4 after 24 weeks FU (σ_24_ = 23.8 Std)
  MCPI: (46.3+26.4) · 2 = 145.4 Std: 108.6

Here, we use the conservative assumption of a perfect correlation between both measurements (W_0_ and W_24_) and calculate Var(MCPI) = 4 · [ σ_0_^2^ + σ_24_^2^ + 2 · σ_0_ · σ_24_]. If the MCPI should be reduced from 132 to 66 assuming a standard deviation (Std) of 110, a total of 46 patients per group are needed in order to assess this difference with a power of 80% on a two-sided 5% significance level.

This clinical study will be analyzed according to the intention-to-treat (ITT, estimand: treatment policy approach) principle. The per protocol (PP) population is a subset of the ITT population and is defined as the group of subjects who had no major protocol violations, received a predefined minimum dose of the treatment and underwent the examinations required for the assessment of the endpoints at relevant, predefined times. The analysis of the PP group will be performed as a sensitivity analysis.

The primary analysis will test a primary endpoint with two components: MCPI and OS. The assessment on the MCPI uses a stratified Mann–Whitney-test. The assessment of the second component OS uses a stratified Cox model. A hierarchical testing will be performed to adjust for the multiple testing implied by the two components of the primary endpoint.

Furthermore, a sensitivity analysis will adjust for unequal observation time and use the difference in pain cumulated over 1 year between both treatment groups. Here, we follow the hypothetical estimand strategy for dealing with intercurrent events, wanting to know what outcomes would have been observed if patients had survived 1 year [29]. In this study, there are several types of intercurrent events (ICEs: like death, drop-out, toxicity), which stay in conflict with a standardized process for longitudinal measurements of pain over a fixed time period. We also take into account the informative drop-out of individual patients triggered by extreme pain.

To avoid bias introduced by ICEs regarding the treatment effect on pain, the concept of joint modelling [30] will be applied. Joint models allow a simultaneous look on time course data of pain and events over time that let the patient drop out and make further measurements of pain impossible. This sensitivity analysis will model time course of pain together with tumor-related events and mortality (PFS, OS). A U-shaped curve models the time course of pain, allowing for a decrease and later increase of pain intensity. The estimator derives from the difference between the areas under the curves (AUCs) specified by the main effects of the joint modelling for the longitudinal pain data over 1 year.

We have no access to documented longitudinal pain data. Therefore, we use a sample size recalculation on unblinded data performed 1 year after the 80th patient was included into the study. This allows sample size adjustments due to unknown treatment effects and variance and allows the review of the actual study data. It also requires strict firewalls to prevent leakage of information about adaptive rules or decisions [31].

### Timelines and responsibilities

Recruitment is planned to start in October 2021 and the recruitment period extends over 2 years, the last patient included in trial will be followed for 6 months. Accordingly, completion of recruitment is expected by October 2023 and the last patient visit by April 2024. First results of the main analysis should be available by end of 2024. The principal investigators are responsible for the project management and study conduct in collaboration with medical staff at every site. The contract research organization is responsible for the safety management (i.e. documentation of adverse events, severe adverse events, reporting) and the monitoring. The statistical plan and data analysis is provided by the Institute for Medical Information Processing, Biometry, and Epidemiology before database closure.

## Discussion

An adequate and long-term control of pain symptoms in patients with mPDAC is an unmet clinical need. Although recent advances in chemotherapy have resulted in a moderate improvement of life expectancy for those patients in good performance state [9–11], local complications due to the primary pancreatic tumor remain a clinical challenge [32, 33] and are accompanied by symptoms, primarily pain. The independent addition of innovative radiotherapy to the most effective systemic therapy in this patient group could therefore lead to an improvement of pain control and quality of life. Seamless integration of radiotherapy into systemic treatment without causing a delay is crucial in this patient group with an aggressive tumor susceptible to rapid systemic progression. This gives the rationale for the delivery of high radiation doses accurately to the target lesion in form of SBRT, given in few outpatient fractions.

There is scarce literature on SBRT in metastatic pancreatic cancer patients and only some retrospective and few prospective data on its effectiveness for pain control of primary pancreatic cancer. One retrospective single-institutional study investigating the role of SBRT in a cohort of metastatic pancreatic cancer patients that evaluated oncological outcome and toxicity of treatment can be found [34], but no details on symptoms or eventual responses to the treatment were given. The tumor was treated with 25–30 Gy in 5 fractions with the prescription isodose line covering at least 95% of the PTV and all patients received concurrent or sequential chemotherapy. Dose constraints to the OAR (stomach, duodenal, and small bowel) were as follows: V30 Gy < 1 cm^3^, V20 Gy < 5 cm^3^, and V10 Gy < 10 cm^3^. The mean PTV was quite large with a volume of 188 cc and a median volume of 147 cc—which is larger compared to that treated with SBRT for LAPC [18]. No grade 3 or greater late toxicities were reported. Another recently published report looked retrospectively at 27 patients with mPDAC and evaluated the effect on abdominal pain, which was present before treatment in 17 patients [35]. SBRT of the primary tumor was given in 1 (median dose of 25 Gy, range: 12.5–25 Gy) or 5 fractions (median dose: 33 Gy, range: 25–40 Gy) and achieved a significant reduction in the mean intensity of pain with an acceptable toxicity profile. Only 2 patients (7%) experienced a grade 3 toxicity and no grade 4 or 5 toxicity has been reported. Another trial looked retrospectively at the effectiveness of SBRT in elderly patients aged ≥ 80 with two of them having stage IV disease [26]. Pain relief has been reported in 75–80% of patients.

This finding of good pain control was confirmed by a recently published systematic review investigating pain relief by SBRT in LAPC [27], with an overall pain relief achieved in 84.9% of patients, but with high heterogeneity of results and complete response rates ranging from 15 to 81.3%. Of note, out of 14 studies in total (7 of prospective and 7 of retrospective design) only four included a minor proportion of metastatic patients [26, 36–38]. Onset of abdominal pain relief was reported by one study and was described to be within 2 weeks of completing treatment [37]. Pain-free survival, also only reported by one study, was at maximum 24 weeks [36]. Even though this important systematic review gives us evidence to use SBRT for the treatment of pancreatic cancer-induced pain it must be stated, that none of the studies reporting on pain relief after SBRT looked at it as a primary endpoint and pain response was not reported in a systematic way.

Overall, there is some evidence to underpin the rationale of a good and durable pain control of primary pancreatic cancer by SBRT in mPDAC. However, in light of the overall limited prognosis and high burden of patients with mPDAC this intervention has to be tested in a prospective randomized trial, which is exactly the purpose of our study.

The treatment should not only be effective in terms of pain control but also accompanied by an acceptable toxicity profile. SBRT of the primary tumor in LAPC with 5 fractions of 6.6 Gy was shown to have a reasonable toxicity profile on a conventional LINAC with 11% late ≥ G2 toxicity [24], which increased to 47% when the treatment was performed in 1 fraction with 25 Gy [39].

In metastatic patients the risk–benefit ratio has to be more beneficial compared to a curative situation, considering the limited prognosis and therefore asking for a focus on QoL. Image-guided radiotherapy is today based on cone-beam CT imaging (CBCT), which does not allow accurate visualization of the pancreas and the OAR in close proximity (duodenum, stomach and small bowel), leading to difficulties to precisely deliver radiation on a daily basis.

Visualization of OAR is more accurate on Magnetic Resonance Imaging, which provides a better soft tissue contrast compared to CT and/or CBCT. Recently developed MR-LINAC hybrid devices allow for MR image-guided radiation therapy (MRgRT) and achieve an improved sparing of OAR [40], which are in close proximity of the pancreatic cancer through better visualization of abdominal soft tissues. Simultaneously, MR-guided adaptive radiotherapy offers the possibility for daily on-table plan adaptation aiming to further improve the therapeutic ratio. Accordingly, in dosimetric studies daily adaptive radiation therapy on a MR-LINAC was shown to enable a better target coverage and superior sparing of OAR at the same time, compared to non-adaptive radiotherapy [40, 41], potentially translating into better tolerance of the treatment and lower risk of late toxicity [42]. Moreover, treatment on a MR-LINAC allows for real-time imaging and tracking of the tumor during treatment. This enables to deliver the treatment using a breath hold technique, which consequently reduces the treatment volume, as the pancreas underlies respiration-induced mobility.

Based on this background, we hypothesize that patients with metastatic pancreatic cancer will benefit from local treatment of the primary tumor by MR-guided SBRT in terms of a long pain control with a simultaneously safe toxicity profile translating into an improvement of quality of life. This will be tested in this prospective, double-arm, parallel group, randomized controlled, international multi-center study aiming to include in total 92 patients. Findings of this trial will provide the basis for the decision whether SBRT should be added to SoC-CHT in patients having a stable disease after initial 8 weeks of systemic treatment.

## Data Availability

Not applicable.

## Abbreviations

mPDAC: Metastasized pancreatic ductal adenocarcinoma
SBRT: Stereotactic body radiotherapy
RFA: Radiofrequency ablation
IRE: Irreversible electroporation
HIFU: High intensity focused ultrasound
NRS: Numeric rating scale
LAPC: Locally advanced pancreatic cancer
SoC-CT: Standard of care chemotherapy
OAR: Organs at risk
MCPI: Mean cumulative pain index
AUC: Area under the curve
ITT: Intention-to-treat
PFS: Progresssion-free survival
OS: Overall survival
ICE: Intercurrent events
